# The effect of reflexology on blood pressure and heart rate in cardiovascular patients: a meta-analysis study

**DOI:** 10.1590/1806-9282.20250059

**Published:** 2025-08-08

**Authors:** Gülşah Çamcı, Betül Bayrak

**Affiliations:** 1 Marmara University, Faculty of Health Sciences, Department of Nursing, Department of Internal Medicine Nursing – İstanbul, Türkiye.; 2 Süleyman Demirel University, Faculty of Health Sciences, Department of Nursing, Department of Internal Medicine Nursing – Isparta, Türkiye.

**Keywords:** Reflexology, Cardiovascular diseases, Blood pressure, Heart rate, Meta-analysis

## Abstract

**OBJECTIVE:**

The aim of the study was to conduct a meta-analysis of studies on the effect of reflexology on blood pressure and heart rate in patients with cardiovascular disease.

**METHOD:**

The study collected data from PubMed, ScienceDirect, MEDLINE, Web of Science, Google Scholar, and Cochrane Library databases. The study had randomized-controlled, non-randomized-controlled, and quasi-experimental designs. Patients aged >18 years with coronary artery disease, coronary angiography, percutaneous coronary intervention, open-heart surgery, or coronary bypass were included. All analyses were performed using Comprehensive Meta-Analysis.

**RESULTS:**

This study included 1,041 patients aged 18–75 years from 12 studies. According to the random-effects model, reflexology significantly reduced systolic blood pressure (95%CI, Hedges’ g=-0.41, Z=-2.27, p=0.02) and diastolic blood pressure (95%CI, Hedges’ g=-0.38, Z=-2.69, p=0.01). However, reflexology did not affect the heart rate (95%CI, Hedges’ g=0.16, Z=-1.54, p=0.12).

**CONCLUSION:**

The results suggest that reflexology has a positive effect on the systolic and diastolic blood pressures of cardiovascular patients, but no significant effect on the heart rate was observed.

## INTRODUCTION

Reflexology is a method applied with special hand and finger techniques to reflex points corresponding to all parts, organs, and systems of the body, located in the ears, hands, and feet. Reflexology is most commonly applied to the feet. Although exact information about where and how reflexology was first applied is not known, it is thought to have emerged in China approximately 5,000 years ago. Murals showing reflexology practiced in the pyramids of Egypt in 2030 BC were found. In the United States, Dr. William Fitzgerald is known to be the first healthcare professional to apply reflexology as the theory of "zone therapy" in 1913^1^. Foot reflexology involves applying controlled pressure using the thumb and index finger to a specific point on the foot corresponding to internal organs, glands, and body parts^2^. Reflexology is believed to improve circulation, energy flow, and stimulate the body's homeostasis regulation. The science of reflexology believes that the potential for healing lies within every individual, and should only help one's healing potential to restore balance and optimize health^2^.

Reflexology is a complementary and alternative therapy widely used to treat various diseases and symptoms, including cardiovascular diseases. Studies have suggested that reflexology can regulate blood pressure, heart rate, and respiration by providing autonomic nervous system modulation, reducing sympathetic nervous system activation, activating the parasympathetic nervous system, and promoting energy flow and balance^3^.

Although some studies have demonstrated the positive effects of reflexology on cardiovascular parameters, there is a lack of sufficient information on the benefits and effectiveness of reflexology in patients with cardiovascular diseases^1,4^. Therefore, this meta-analysis aimed to determine the overall effect of studies examining the effect of reflexology on blood pressure and heart rate in patients with cardiovascular diseases.

## METHODS

### Purpose of the research

The aim of this study was to conduct a meta-analysis of the effects of reflexology on blood pressure and heart rate in patients with cardiovascular disease.

### Data scan

The research strategy of the study involved using key terms according to the PICOS (population, intervention, comparator, outcomes, and study design). The study employed Medical Subject Headings (MeSH) terms or keyword combinations, encompassing reflexology, blood pressure, heart rate, heart, coronary, cardiovascular, and physiological parameters. The study included randomized-controlled, non-randomized-controlled, and quasi-experimental studies conducted in English with no date restrictions. The two authors independently searched for sources in PubMed, ScienceDirect, Medline, Web of Science, Scopus, Google Scholar, and Cochrane Library databases, and duplicates were removed using the EndNote program. The titles and abstracts of the remaining articles were examined, and studies that did not meet the inclusion criteria were excluded. Data from the included studies were entered into an Excel spreadsheet.

Population: The study included patients aged >18 years with coronary artery disease, coronary angiography, percutaneous coronary intervention, open-heart surgery, or coronary bypass. Patients in the experimental group received reflexology intervention, whereas those in the control group received routine treatment.

Intervention: The experimental group received hand or foot reflexology, and blood pressure and heart rate were measured before and after the intervention.

Comparison: The control group received routine treatment in the hospital.

Outcomes: The primary results of the meta-analysis were the mean and standard deviation of the blood pressure and heart rate.

Study design: This study included randomized, controlled, non-randomized-controlled, and quasi-experimental studies conducted in English.

### Search outcome

A literature search was performed according to PRISMA guidelines, and 375 resources were recorded in the EndNote program from the PubMed (28), ScienceDirect (12), Medline (49), Web of Science (88), Scopus (105), Google Scholar (43), and Cochrane Library (50) databases. Advanced scanning was performed, and 43 duplicate studies were removed, leaving 332 articles. The titles and abstracts of 332 articles were reviewed, and 314 studies were excluded because they were non-cardiovascular diseases, review articles, or included individuals under the age of 18 years. The remaining 18 articles were evaluated; one study was proceeding, and the language of three studies was not English, leaving 14 articles. Notably, 2 of the 14 studies were excluded due to lack of data, leaving 12 studies for the meta-analysis.

### Statistics

All analyses were performed using the Comprehensive Meta-Analysis software (CMA, version 3.0). The Hedges’ g effect size, which measures the standardized mean difference in each study, was calculated by entering the mean and standard deviation of the systolic, diastolic, and heart rates of the reflexology and control groups in the studies. For heterogeneity, the Cochrane Q (p<0.05 value indicates heterogeneity) and l^2^ (25% interpreted as low, 50% moderate, and 75% high heterogeneity) statistics were examined^5^. A common standard effect size was found according to the random-effects model. Publication bias funnel plots were analyzed by visual inspection, and the Egger test and Begg and Mazumdar rank correlation were used to assess publication bias. If these tests showed significant results, there was publication bias.

## RESULTS

Characteristics of included studies and patients

A total of 12 studies^6-17^, 10 of which were RCTs, were included in the meta-analysis, and the total number of participants was 1,041. The ages of the patients in these studies ranged from 18 to 75 years. Notably, 10 of these studies were on foot reflexology, and the remaining two studies were on hand reflexology^7,9^, and aimed to determine the effectiveness of reflexology on blood pressure and heart rate. Summary data of the existing studies are shown in Table 1.

**Table 1:** Summary of included studies.

Author	Design	SBP result	DBP result	HR result
Abbaszadeh et al.^17^	RCT	**p=0.006**	**p=0.001**	p=0.074
Bahrami et al.^16^	RCT	**p=0.01**	p=0.37	**p=0.01**
Ebadi et al.^15^	RCT	p=0.06	p=0.37	p=0.48
Elsayed et al.^14^	Semi-experimental	**p<0.0005**	**p<0.0005**	**p<0.0005**
Hashemzadeh et al.^13^	RCT	**p=0.001**	p=0.955	**p=0.003**
Kandemir et al.^12^	Non-RCT	p=0.572	p=0.717	p=0.805
Khalili et al.^10^		**p=0.02**	**p=0.03**	p=0.082
Khaledifar et al.^11^	RCT	**p<0.001**	**p=0.002**	**p<0.001**
Mei et al.^9^	RCT	p=0.968	p=0.857	p=0.774
Moeini et al.^8^	RCT	**p=0.012**	**p=0.012**	p>0.05
Rahmani et al.^7^	RCT	p=0.47	p=0.84	p=0.90
Saber Mohamed et al.^6^	RCT	p=0.074	p=0.729	p=0.995

SBP: systolic blood pressure; DBP: diastolic blood pressure; HR: heart rate; RCT: randomized controlled study. Statistically significant values are indicated in bold (p<0.05).

### Heterogeneity of studies

**Figure 1:**
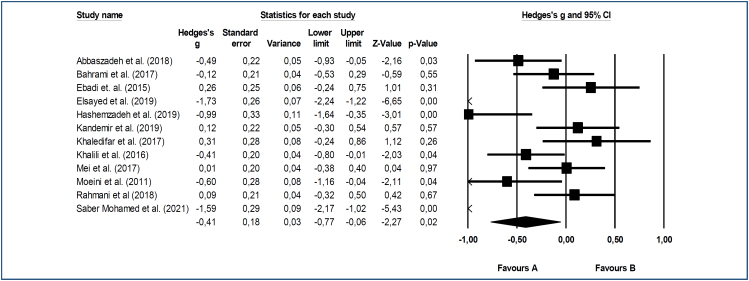
Forest graph of the effect of reflexology on systolic blood pressure.

**Figure 2:**
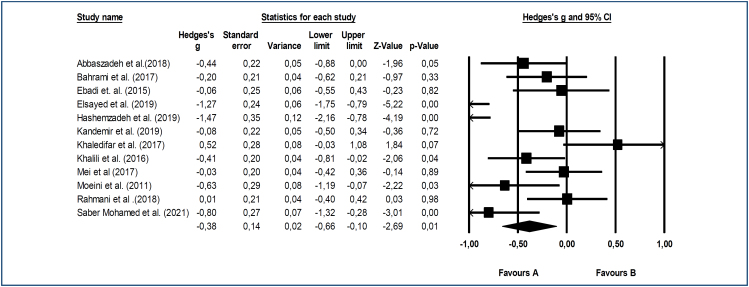
Forest graph of the effect of reflexology on diastolic blood pressure.

In this meta-analysis, post-test results of both the reflexology and control groups were used to analyze the effect of reflexology on blood pressure and heart rate, and the effect size results of the differences were calculated. Heterogeneity of the studies included in the analysis was determined beforehand. When the results were examined, it was observed that the Q statistic results of all three analyses were significant (SKB=0.000, DKB=0.000, KH=0.000). The heterogeneity levels were determined to be over 75% (high heterogeneity) for SBP and DBP, and over 50% (moderate heterogeneity) for heart rate. Accordingly, we estimated the right effect size using the random-effects model. The effect size calculated using the random-effects model in the study and the weights of the studies in the meta-analysis are shown in Figures 1 and 2.

### Effect of reflexology on blood pressure and heart rate

Of the 12 studies included in the meta-analysis, a total of 442 subjects were included in the reflexology intervention group. In six of these studies, namely five RCTs with a total of 205 patients and one quasi-experimental study, reflexology application was found to have a negative and significant effect on systolic blood pressure^8,10,11,13,14,17^. In all of these studies, the reflexology type was foot reflexology. The meta-analysis according to the random-effects model showed that reflexology significantly reduced SBP {confidence interval [CI] 95% Hedges’ g=0.41 (medium effect size)}, and there was a statistically significant difference between the reflexology and control groups (Z=-2.27, p=0.02) (Figure 1).

In five of these studies—four RCTs and one quasi-experimental study with a total of 185 patients—reflexology application was found to have a significant negative effect on diastolic blood pressure^8,10,11,14,17^. In all of these studies, the reflexology type was foot reflexology. A meta-analysis based on the random-effects model showed that reflexology significantly reduced DBP {confidence interval [CI] 95% Hedges’ g=0.38 (medium effect size)}, and there was a statistically significant difference between the reflexology and control groups (Z=-2.69, p=0.01) (Figure 2).

It was found that reflexology practice, which included only four of the studies (65 people), had a significantly negative effect on heart rate^11,13,14,16^. In the meta-analysis performed according to the random-effects model, reflexology had no effect on heart rate (confidence interval [CI] 95% Hedges’ g=0.16) and showed no statistically significant difference between the reflexology and control groups (Z=-1.54, p=0.12).

### Publication bias

A funnel plot indicated that the study results were symmetrical. For SBP, the Egger test of the intercept was −8.00775 (one-tailed, p=0.051) and Kendall τ was −0.272 in the Begg and Mazumdar rank correlation (two-tailed, p=0.217). For DBP, the Egger test of the intercept was −5.447 (one-tailed, p=0.082) and Kendall τ was −0.333 in the Begg and Mazumdar rank correlation (two-tailed, p=0.131). For HR, the Egger test of the intercept was −5.769 (one-tailed, p=0.031) and Kendall τ was −0.454 in the Begg and Mazumdar rank correlation (two-tailed, p=0.046).

## DISCUSSION

Lowering the blood pressure and heart rate is crucial for individuals with cardiovascular disease to prevent cardiac events. High blood pressure and heart rate can lead to sympathetic overload, causing coronary narrowing and peripheral resistance. Therefore, controlling blood pressure and heart rate is the primary condition for disease management and preventing cardiac risk^18^. Among the various non-pharmacological methods such as music therapy and acupuncture, reflexology has shown promise in lowering blood pressure and heart rate^19-21^. However, the evidence from studies in which reflexology intervention was applied is insufficient.

This meta-analysis aimed to evaluate the effect of reflexology as an alternative treatment option for blood pressure and heart rate in individuals with cardiovascular disease. We analyzed 12 studies that met the inclusion criteria. Our systematic review showed that five RCTs and one quasi-experimental study had significant reductions in SBP in the reflexology intervention group^8,10,11,13,14,17^. The same five studies, except for Hashemzadeh et al., also had significant and positive results for DBP (Table 1)^8,10,11,14,17^. The remaining seven studies had no significant results. Our meta-analysis revealed that reflexology had a moderately reducing effect on SBP and DBP (Figures 1 and 2). However, there was a significant difference in heart rate values between the reflexology and control groups in only four studies included in the meta-analysis (Table 1)^11,13,14,16^. This meta-analysis showed that reflexology had no effect on heart rate.

Our results are consistent with those of another meta-analysis^4^ that demonstrated the positive effects of reflexology on lowering blood pressure. In total, 13 RCTs, all of the foot reflexology studies performed in the meta-analysis analyzed by Jing et al., were included, and no specific patient group was selected. In another systematic review, positive results of reflexology on blood pressure and cardiac parameters were reported. However, no specific patient group was selected in this study, and only three articles were included^22^. In yet another systematic review, only three studies were included and showed positive results of reflexology on parameters such as stress and fatigue, but found no effect on blood pressure and heart rate^23^. Another systematic review and meta-analysis reporting positive results of reflexology on anxiety considered its positive effects on cardiac parameters as secondary outcomes only, as a narrative synthesis^24^.

None of the studies included in this meta-analysis revealed any side or harmful effects of reflexology practice. However, further studies with larger sample sizes and long-term follow-up are needed to provide more reliable evidence on the effects of reflexology on blood pressure and heart rate in individuals with cardiovascular disease.

## CONCLUSION

In conclusion, this meta-analysis suggests that reflexology can moderately reduce blood pressure but has no effect on heart rate in individuals with cardiovascular disease. However, the small sample size and lack of high-quality studies limit the conclusions that can be drawn. Further randomized controlled trials are necessary to determine the effects of reflexology on cardiovascular parameters. Nevertheless, reflexology can be considered a complementary intervention to lower blood pressure in individuals with coronary artery disease, those undergoing coronary angiography, patients undergoing percutaneous coronary intervention, and individuals undergoing open-heart surgery or coronary bypass.

## Data Availability

The datasets generated and/or analyzed during the current study are available from the corresponding author upon reasonable request.
